# Genome-Wide Analysis of the Expression of Circular RNA Full-Length Transcripts and Construction of the circRNA-miRNA-mRNA Network in Cervical Cancer

**DOI:** 10.3389/fcell.2020.603516

**Published:** 2020-11-24

**Authors:** Tianyi Xu, Xiaofeng Song, Yulan Wang, Shilong Fu, Ping Han

**Affiliations:** ^1^Department of Biomedical Engineering, Nanjing University of Aeronautics and Astronautics, Nanjing, China; ^2^Department of Obstetrics and Gynecology, The First Affiliated Hospital of Nanjing Medical University, Nanjing, China

**Keywords:** cervical cancer, circular RNA, transcriptional regulation, full-length transcripts, network

## Abstract

Increasing evidence suggests that circular RNA (circRNA) plays an important role in tumorigenesis by regulating gene expression at the transcriptional and post-transcriptional levels. Alternative splicing events permit multiple transcript isoforms of circRNA to be produced; however, changes in the expression of circRNA full-length transcripts in cervical cancer remain unclear. Here, we systematically explored the dysregulation circRNA full-length transcripts and constructed an improved circRNA-miRNA-mRNA regulatory network to provide potential biomarkers and possible treatment targets in cervical cancer. We identified 9359 circular full-length transcripts from RNase R-treated RNA-seq data in cervical cancer, of which 353 circular full-length transcripts were significantly differentially expressed (DE) between the tumor and normal group. A total of 881 DE mRNA transcript isoforms were also identified from total RNA-seq data in cervical cancer, of which 421 (47.8%) transcript isoforms were up-regulated, and 460 (52.2%) transcript isoforms were down-regulated in tumor samples. Two circRNA-miRNA-mRNA competitively regulated networks, including 33 circRNA transcripts, 2 miRNAs, and 189 mRNA transcripts were constructed. Three genes (*COPE*, *RAB3B*, and *TFPI*) in the network were significantly associated with overall survival (*P* < 0.05), which indicated that these genes could act as prognostic biomarkers for patients with cervical cancer. Our study revealed genome-wide differential expression of full-length circRNA transcripts and constructed a more accurate circRNA-miRNA-mRNA network at the full-length transcript expression level in cervical cancer. CircRNA may thus be involved in the development of cervical cancer by regulating the expression of *COPE*, *RAB3B*, and *TFPI*. However, the specific regulatory mechanism in cervical cancer requires further study.

## Introduction

Cervical cancer is the fourth most common type of cancer diagnosed in women (Bray et al., 2018) and the fourth main cause of cancer mortality in women; cervical cancer thus poses a serious threat to the health of women worldwide. Statistics from the Global Cancer Annual Report from 2018 revealed that there were approximately 570,000 new cases of cervical cancer and that approximately 311,000 cancer patients died of cervical cancer (Bray et al., 2018). Cervical cancer accounts for 80% of all cancers attributable to human papillomavirus (HPV) infection. According to statistics released by the China Cancer Center in 2015, the incidence of cervical cancer in China showed consistent annual increases from 2000 to 2011, and the age of onset shifted to younger ages (Chen et al., 2016). Cervical cancer is the sixth most common cause of female malignant tumors, accounting for 6.25% of cases. In China, there are approximately 100,000 new cases of cervical cancer and 30,000 deaths from cervical cancer annually. Current treatment methods for cervical cancer include surgery, radiotherapy, chemotherapy, and comprehensive treatment. Cervical adenocarcinoma is prone to lymphatic metastasis in the early stage, and the prognosis is relatively poor. Patients with advanced cervical cancer have lost the opportunity for surgical resection because of tumor metastasis and cannot bear the side effects of radiotherapy and chemotherapy (Cohen et al., 2019). There is thus a need to explore the internal mechanism of cervical cancer and identify new therapeutic targets.

With the development of next-generation sequencing technology and the integrated analysis and application of multiple omics data in recent years, non-coding RNA (ncRNA), such as microRNA (miRNA), long non-coding RNA (lncRNA), piRNA, and snoRNA, has been shown to be involved in the biological processes of various cancers. Researchers have also recently reported a new type of non-coding RNA with a special structure—circular RNA (circRNA) (Memczak et al., 2013; Salzman et al., 2013; Jeck and Sharpless, 2014; Li et al., 2015; Xu et al., 2017). CircRNAs are a large class of covalently closed transcripts that do not have a 5’-end cap structure and a 3’-end polyadenylated (PolyA) tail; thus, they are not easily degraded by exonucleases and are structurally stable and tissue specific. Previous studies have shown that circRNA has the potential to compete with endogenous RNA. CircRNA can act as a “sponge” of miRNAs by naturally isolating and competitively inhibiting the activity of miRNA, thereby indirectly regulating miRNA target genes (Hansen et al., 2013a; Liu et al., 2016; Xie et al., 2016; Yu et al., 2016; Han et al., 2017; Zhang et al., 2017). Like lncRNA and miRNA, circRNA also plays an important role in cervical cancer (Tornesello et al., 2020). For example, Tang et al. found that hsa_circ_0000515 can promote the development of cervical cancer (Tang et al., 2019). Specifically, hsa_circ_0000515 acted as a competitive endogenous RNA that specifically bound to miR-326 and inhibited its expression, thereby increasing the expression level of the target gene ETS transcription factor ELK1 (*ELK1*). The enhancement of *ELK1* expression led to the enhancement of cervical cancer cell proliferation and invasion ability but repressed cell apoptosis and autophagy. Similarly, Song et al. found that reducing the expression of hsa_circRNA_101996 can significantly suppress the proliferation, migration, and invasion of cervical cancer cells (Song et al., 2019). Specifically, hsa_circRNA_101996 can act as a sponge of miR-8075 and down-regulate its expression level, thereby regulating miR-8075’s target gene *TPX2* and affecting the occurrence and development of cervical cancer. Zhao et al. also reported that the virus-encoded circular RNA-circE7 in HPV is preferentially located in the cytoplasm and that circE7 can be modified by N6-methyladenosine (m6A) and translated to produce E7 oncoprotein. The increased expression of E7 oncoprotein contributes to certain transforming activities of HPV cells (Zhao J. et al., 2019). In sum, both circRNA in the human body and circRNA produced by viruses play an important role in the biological processes of cervical cancer.

**FIGURE 1:**
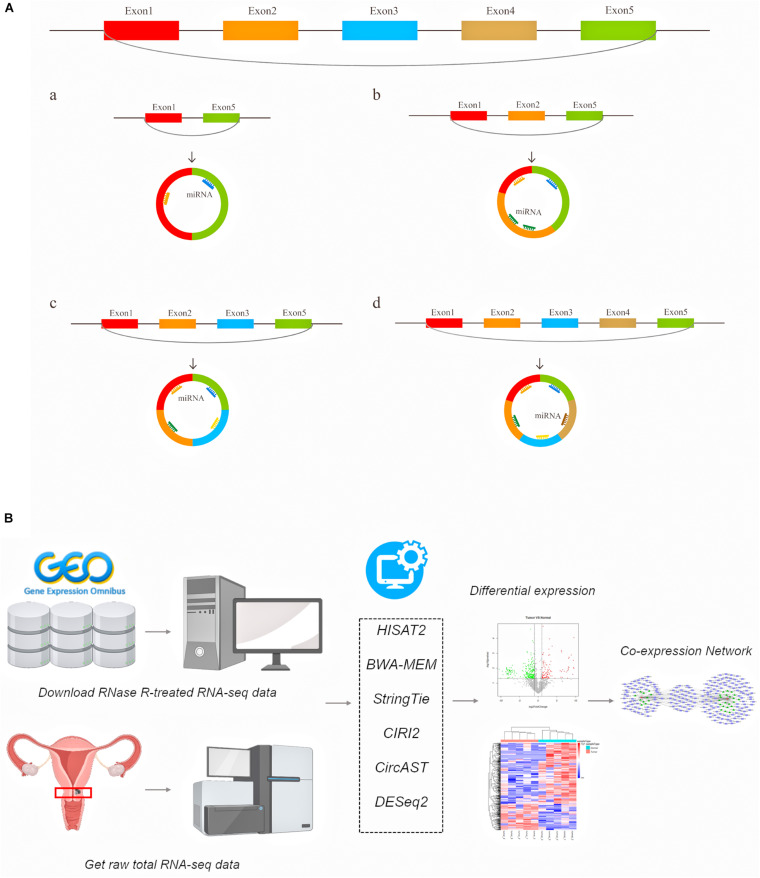
The schematic diagram of generated different circular RNA full-length transcripts in cervical cancer **(A)** and the flowchart of analyses in this study **(B)**.

A large number of circular RNAs have been identified in multiple species to date, and a small number of circRNAs have been shown to be involved in gene regulation (Zhao J. et al., 2019), growth and development (Kristensen et al., 2018), and the innate immune response (Li X. et al., 2017), and these circRNAs play an important role in various types of diseases, including cancer (Hirsch et al., 2017; Li P. et al., 2017; Piwecka et al., 2017; Yu et al., 2017; Chen et al., 2019; Vo et al., 2019). Existing circRNA-related research primarily focuses on using the total read counts aligned to back-splice junctions to estimate circRNA expression. However, the internal structure of alternative splicing circRNA transcripts and the expression of full-length circRNA transcripts in cancer remain unclear. Previous studies have suggested that alternative splicing events can cause circRNA to produce multiple transcript isoforms (Gao et al., 2016; Zhang X.-O. et al., 2016; Wu et al., 2019). Our understanding of the functions of specific circRNAs, including the evolutionary characteristics of circular RNAs between species and the differences in circular RNA transcripts between individuals and groups, remains limited. Moreover, the binding sites of circRNAs and protein-coding potentials also require information on the entire lengths of circRNAs. The diagram of this mechanism is shown in Figure 1A. For example, there are circRNAs derived from exon regions that contain five exons. Through different alternative splicing methods, circRNA can produce 4 full-length circRNA transcript types with the same back splicing site but with different numbers of exons (Figure 1A a–d).

Here, we used a series of bioinformatics tools to systematically explore the differentially expressed (DE) full-length circRNA transcripts and more precisely predict the regulation target sites of circRNA in cervical cancer (Figure 1B). Generally, our aim was to aid the search for potential biomarkers and possible treatments for cervical cancer.

## Materials and Methods

### Public Datasets and Patient Sample Collection

We retrieved the number GSE107472 (Li et al., 2019)from the Gene Expression Omnibus database of the National Center for Biotechnology Information to determine the differential expression of full-length circRNA transcripts in cervical cancer. The public dataset included the RNA-seq data of 5 cervical cancer patients with primary tumors and matched adjacent normal samples, which were processed by ribonuclease R (RNase R) to remove linear RNAs and ribosomal RNA (rRNA).

To further verify and explore the function of differential full-length circRNA transcripts, we collected 4 pairs of samples from the primary tumors and adjacent normal tissue of cervical cancer patients from The First Affiliated Hospital with Nanjing Medical University to characterize differences in the abundances of mRNA transcripts. Sample collections were approved by the Human Research Ethics Committee of The First Affiliated Hospital with Nanjing Medical University (No.2020-SR-387), and permission from the cervical cancer patients was also obtained.

### Total RNA Extraction and Sequencing

First, we cut the clinical samples into small pieces and added TRizol reagent (Life Technologies) to homogenize samples with a homogenizer. The total RNA extraction kit (Tiangen, China) was used to extract the total RNA from each sample per the manufacturer’s instructions. Whole transcriptome libraries were constructed using the TruSeq Ribo Profile Library Prep Kit (Illumina, United States) per the manufacturer’s protocol. Briefly, 10 mg of total RNA was depleted of rRNA with an Illumina Ribo-Zero Gold kit and purified for end repair and 5’-adaptor ligations. Next, reverse transcription was performed with random primers containing 3’ adaptor sequences and randomized hexamers. Finally, cDNA fragments of 250–400 bp were purified and amplified by 15–20 cycles of PCR. The libraries were subjected to 150 nt paired-end sequencing with an Illumina Hiseq4000 system (BGI, China). At least 45 million read pairs were generated for each library, and adapters were removed with Cutadapt (Martin, 2011) to obtain clean reads.

The raw sequence data reported in this paper have been deposited in the Genome Sequence Archive (Wang et al., 2017) in National Genomics Data Center, Beijing Institute of Genomics (China National Center for Bioinformation), Chinese Academy of Sciences, under accession number HRA000394 that are publicly accessible at http://bigd.big.ac.cn/gsa-human.

### Identification of Dysregulated circRNA Full-Length Transcripts and Linear mRNA Transcripts

Ten RNase R-treated RNA-seq samples (GSE107472) and 8 total RNA-seq samples were used to identify dysregulated circRNA full-length transcripts and linear mRNA transcripts, respectively. First, the raw sequence reads were cleaned by trimming the low-quality bases (Q < 20). The cleaned RNA-seq reads were then mapped to the human reference genome (GRCh37/hg19, UCSC Genome Browser) by HISAT2 (Kim et al., 2015) (version 2.1.0) and BWA-MEM (Heng, 2013) software. These two tools are capable of detecting canonical splicing events. Next, CIRI2 (Gao et al., 2018) (version 2.0.6) was used to identify circRNAs in each sample by recognized the back-splicing reads (≥ 2). CIRI2 is an algorithm based on a multiple-seed-matching strategy. Specifically, it extracts seed sequences in descending order of length for genomic regions with lower comparison quality and quickly matches them with the front and rear flanking genomic regions. At the same time, a maximum likelihood estimation model was established to determine the actual source of the seed sequence and eliminate interference from linear transcripts or splicing byproducts, thereby greatly improving the accuracy of circular RNA molecular recognition. Finally, StringTie (Pertea et al., 2015) (version 1.3.4d) and CircAST (Wu et al., 2019) were used to assemble linear mRNA transcripts and full-length circRNA transcripts, respectively. CircAST is a tool for assembling and quantifying full-length circRNA transcripts in RNase R-treated RNA-seq datasets based on graph theory and the minimum path coverage algorithm. To evaluate the relative expression of mRNA transcripts between tumor samples versus adjacent normal samples, DESeq2 (Love et al., 2014), an R package for differential expression analysis based on negative binomial generalized linear models, was used with the cutoff values [adjusted *p-*value < 0.05, | log2(fold change)| > 1]. Read counts mapped to the sequence of mRNA transcripts were used. Student’s t-tests were used to determine DE full-length circRNA transcripts between tumor and adjacent normal samples [*P* < 0.05, | log2(fold change)| > 1]. The relative expression level of full-length circRNA transcripts in fragments per kilobase per million mapped reads (FPKM) was also used.

### Construction of the circRNA-miRNA and ceRNA Network

To predict the relationship between the DE full-length circRNA transcripts and dysregulated miRNAs and the relationship between the dysregulated miRNAs and its target mRNA transcripts, miRanda (Betel et al., 2010) (August 2010 Release) and TargetScan (Agarwal et al., 2015) (Release 7.1) pipelines were used to predict the circRNA-miRNA interaction and circRNA-miRNA-mRNA competitively regulated network. The sequences and the annotation of miRNAs were obtained from the miRbase (Kozomara et al., 2019) database. In the miRanda pipeline, we set parameters with match scores higher than 150 and minimum free energy less than -20 kcal/mol to improve the reliability of our prediction. The visualization software Cytoscape (Shannon et al., 2003) (version 3.7.1) was used to display the above networks.

### Integrated Functional Enrichment Analysis

The corresponding parental genes of full-length circRNA transcripts and the significantly dysregulated mRNA transcripts in the ceRNA network were both used for the functional enrichment analysis. Gene Ontology (GO) enrichment analysis (including Biological Processes, Cellular Components, and Molecular Function) and Kyoto Encyclopedia of Genes and Genomes (KEGG) pathways were performed using the WEB-based Gene Set Analysis Toolkit (WebGestalt) (Liao et al., 2019) with default parameters.

### Evaluation of Overall Survival for Genes

We utilized the Gene Expression Profiling Interactive Analysis (GEPIA) database (Tang et al., 2017) to explore the survival curves. GEPIA captures the relationship between patient survival information and gene expression based on TCGA datasets. We selected cervical squamous cell carcinoma (CESC) and input the DE genes corresponding to DE mRNA transcripts in the network to explore the association between DE genes and overall survival (OS). The genes with *P* < 0.05 were considered as critical genes.

## Results

### Identification of Full-Length circRNA Transcripts in Cervical Cancer

**FIGURE 2:**
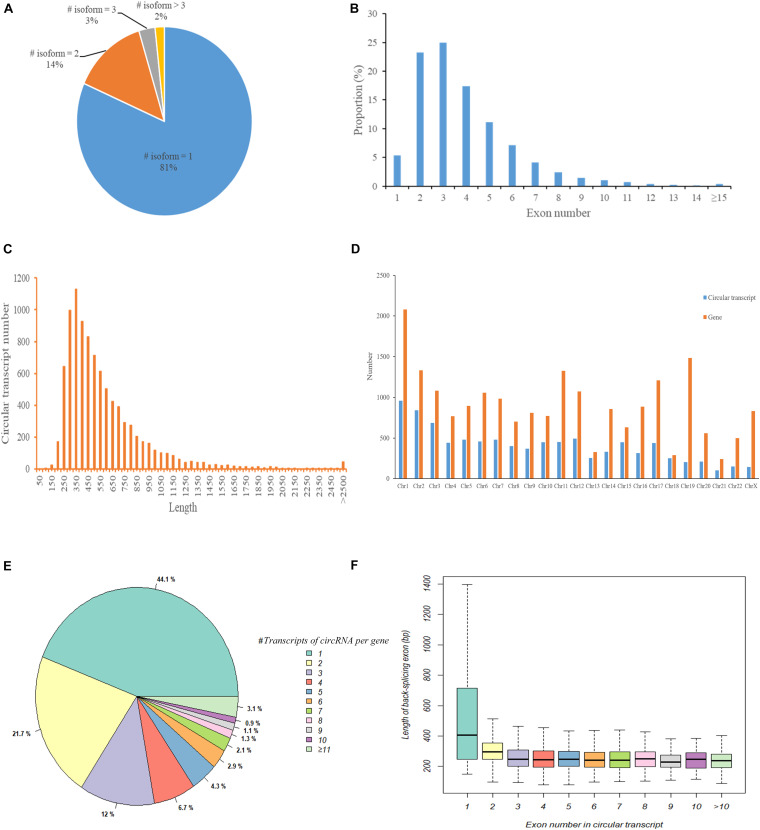
The characteristics of circular RNA full-length transcripts in cervical cancer. **(A)** The distribution of full-length transcripts in circular RNA; **(B)** The distribution of exon numbers in circRNA full-length transcripts; **(C)** The length distribution of circRNA full-length transcripts; **(D)** The distribution of circRNA transcript number and gene number in different chromosomes; **(E)** The distribution of circRNA full-length transcript number produced by parental gene; **(F)** The length distribution of circ-exons contained in circular full-length transcripts composed of different number of exons.

We first used CIRI2 to identify circRNAs in the tumors of 5 cervical cancer patients and adjacent normal samples. A total of 14,125 circRNAs with different back splicing sites were identified. According to the chromosomal location of circRNAs, we concluded that 82.8% of circRNAs were derived from exonic regions and 10.7% of circRNAs were derived from intronic regions; only 6.5% of circRNAs were derived from intergenic regions. Next, we used CircAST software to assemble and quantify the full-length transcripts of the circRNA derived from exons in cervical cancer. We successfully constructed a total of 9359 different full-length circRNA transcripts in primary tumor and adjacent normal tissues in cervical cancer. We found that most circRNAs (81%) had only one circular transcript isoform and that 14% and 2% of circRNAs had two and three circular transcript isoforms, respectively (Figure 2A). Approximately 2% of circRNAs contained more than 3 circular transcript isoforms. Although most circRNA have only one circRNA transcript isoform in cervical cancer, alternative splicing can occur inside circRNA, resulting in the formation of multiple different full-length circRNA transcripts.

Next, we analyzed the internal composition of these full-length circRNA transcripts in cervical cancer. Approximately 71% of the full-length circRNA transcripts had less than 4 internal exons, and nearly half of the circular transcripts were primarily composed of two exons (23.27%) or three exons (24.96%) (Figure 2B). We also found that there were 32 (0.34%) full-length circRNA transcript isoforms with more than 14 exons, of which circSYNE2 (chr14:64529469-64612941) had the most exons (composed of 35 exons). The circRNA transcripts derived from exons in cervical cancer were not particularly long, as most of the full-length circRNA transcript isoforms (5857/9359, 62.58%) were between 250–550 bp (Figure 2C). Transcripts 300–350 bp in length accounted for the highest proportion (12.07%, 1130/9359), and only 0.48% of the transcripts were longer than 2500 bp. We statistically analyzed the number of circRNA transcripts and the number of genes in different chromosomes (Figure 2D) and also compared the number of circular transcripts with the number of linear transcripts and their distribution in chromosomes (Supplementary Table S1). We found that the number of full-length circRNA transcripts was lower than the number of genes on each chromosome, but the lowest variance in gene and transcript numbers was observed on chromosome 18 (total of 252 circRNA transcripts and 289 genes), and the highest variance in gene and transcript numbers was on chromosome 19 (total of 205 circular transcripts and 1485 genes) (Figure 2D). Although the number of full-length circRNA transcripts on the chromosome was much lower than the number of corresponding linear transcripts of parental genes, the ratio of circular transcripts to linear transcripts was relatively stable and was mostly concentrated around 5% (Supplementary Table S1). Chromosome 19 had the lowest proportion (1.61%), whereas chromosome 13 had the highest proportion (8.44%). Figure 2E shows the number of full-length circular transcripts produced by the parental genes of circRNAs. We found that most of the parental genes (77.8%) produced less than 4 full-length circular transcripts in cervical cancer, of which 44.1% genes produced only one full-length circular transcript, 21.7% genes produced two full-length circular transcripts, and 12.0% genes produced three full-length circular transcripts; 3.1% genes could produce more than 10 full-length circular transcripts. Figure 2F shows the length distribution of circ-exons contained in full-length circular transcripts composed of different numbers of exons. The length of circRNA transcript isoforms composed of one exon had a median value of 404.5 bp, which was significantly higher than that of circular transcripts composed of other numbers of exons (median value was approximately 248.1 bp, *p* < 0.05).

### Analysis of DE Full-Length circRNA Transcripts in Cervical Cancer

**FIGURE 3:**
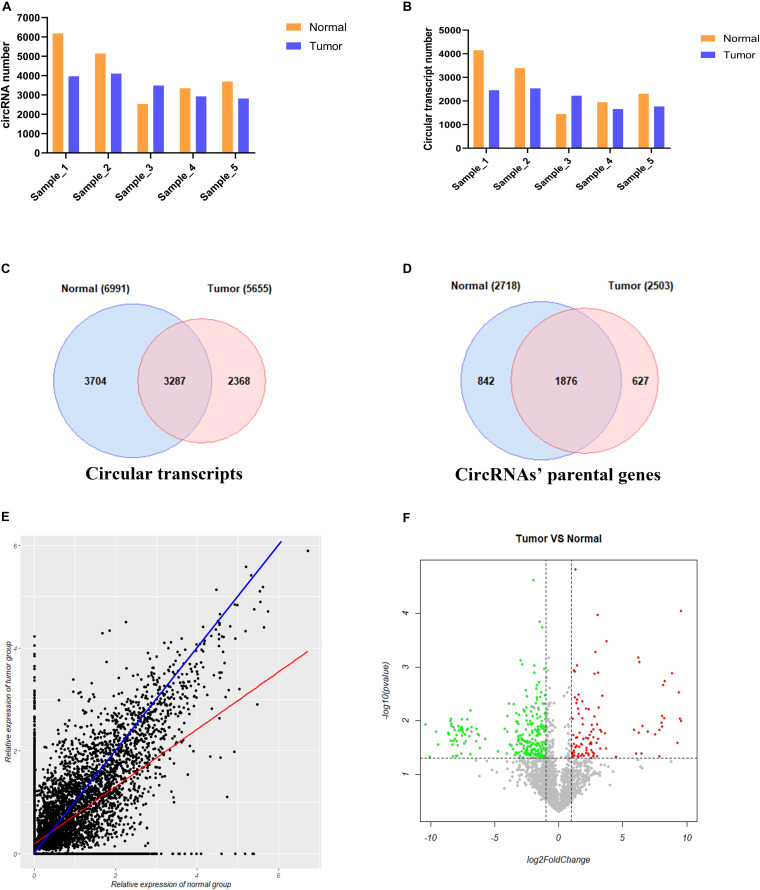
Analysis of circRNA full-length transcripts expression in tumor and adjacent normal tissues in cervical cancer. **(A)** Quantitative analysis of circRNA in tumor and adjacent normal tissues among different patients; **(B)** Quantitative analysis of circRNA full-length transcripts in tumor and adjacent normal tissues among different patients; **(C)** Statistical analysis of the number of circRNA full-length transcripts in tumor and adjacent normal tissues; **(D)** Statistical analysis of the number of parental genes producing circular RNA full-length transcripts in tumor and adjacent normal tissues; **(E)** Analysis of the expression of circular full-length transcript in tumor and adjacent normal tissues. The blue line represents the same transcript expression between the two groups, and the red line represents a straight line fitted according to the relative expression distribution of all circular transcripts in two groups; **(F)** Volcano plot showing differentially expressed circRNA full-length transcripts in cervical cancer. Red points and green points represent significantly up-regulated and down-regulated circRNA full-length transcript isoforms, respectively.

We used CIRI2 software to identify the circRNAs in the tumors of 5 cervical cancer patients and adjacent normal samples. A total of 6183, 5140, 2532, 3346, and 3692 circRNAs were detected in each sample of the normal group, and 3961, 4102, 3483, 2927, and 2813 circRNAs were detected in the corresponding tumor group samples (Figure 3A). These circRNAs were supported by at least two reads that spanned the back-splicing site. We then used the CircAST software to assemble the full-length transcripts of circRNAs identified in each sample. A total of 4148, 3391, 1445, 1949, and 2308 circRNA transcripts were successfully assembled in each sample of the normal group; in the corresponding tumor group, 2456, 2535, 2223, 1661, and 1768 circRNA transcripts were successfully assembled (Figure 3B). Thus, with the exception of patient No. 3, the number of circRNA and the number of full-length circRNA transcripts in the tumor sample were lower than that of adjacent normal samples in cervical cancer. Individual differences between patients may explain the higher number of circRNA and full-length circular transcripts in the tumor sample of patient No. 3 relative to the adjacent normal sample.

Next, we compared the full-length transcripts of circRNA in each sample of the cancer group and the normal group. We found that 6991 and 5655 full-length circRNA transcripts were observed in the tumor group and adjacent normal group, respectively. Among them, 3704 (53.0%) specific full-length circular transcripts were identified in the cancer group, and 2368 (41.9%) specific full-length circular transcripts were successfully assembled in the normal group; there were 3287 full-length circular transcripts expressed in both groups (Figure 3C). Next, we analyzed the parental genes corresponding to the full-length circRNA transcripts between the two groups (Figure 3D). A total of 2718 and 2503 genes were found to correspond to the full-length circRNA transcripts in the tumor and normal group, respectively, of which 842 (31.0%) parental genes were only expressed in the tumor group, 627 (25.0%) parental genes were only recognized in the adjacent normal group, and 1876 genes were expressed in both groups (Figure 3D). To analyze differences in the expression level of the same full-length circRNA transcripts between the two groups, we normalized the expression level of each circular transcript obtained by the CircAST software according to formula 1 and used the relative expression level as estimated by FPKM for further analysis. Formula 1:

$$\text{Relative}\,\mathrm{expression}=\log_{2}\,\left(\mathrm{expression}+1\right)$$

Most of the data points were distributed on both sides of the blue line (Figure 3E), which means that the expression levels of most full-length circRNA transcripts were not significantly different between the cancer group and the adjacent normal group. The blue line represents the same transcript expression level between the two groups, and the red line represents a straight line fitted according to the distribution of the relative expression of all of the circular transcripts in the two groups. The red line was below the blue line, indicating that the differential full-length circular transcripts tended to be highly expressed in the adjacent normal group but weakly expressed in the cancer group. Thus, the number of down-regulated expressed full-length circRNA transcripts was higher than the number of up-regulated expressed full-length circRNA transcripts in tumors.

**FIGURE 4:**
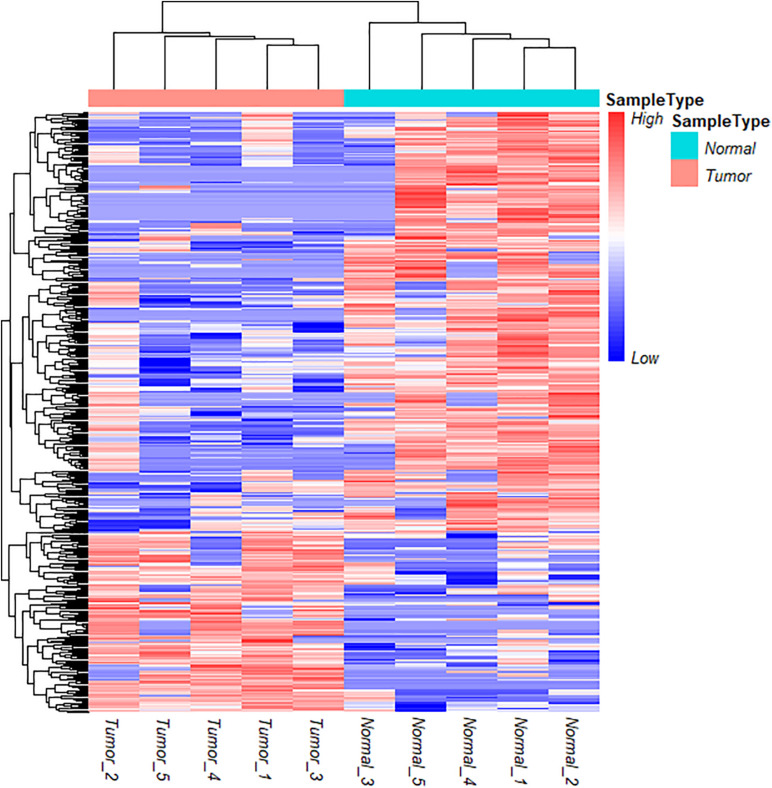
Cluster analysis of circRNA full-length transcripts with significant difference expression in tumor and adjacent samples in cervical cancer.

To further understand the role and potential functions of full-length circRNA transcripts in cervical cancer, we first defined full-length circRNA transcripts expressed in at least 4 samples as high-confidence full-length circRNA transcripts. After filtering, we obtained a total of 2133 high-confidence full-length circRNA transcript isoforms; next, Student’s t-tests were used to determine the DE full-length circRNA transcripts in tumor and adjacent normal samples. Statistical analysis revealed that 353 full-length circular transcripts were significantly DE between the tumor and normal group (| log2 (fold change)| > 1, adjusted *p*-value < 0.05). Among them, 107 circular transcripts (30.3%) were significantly up-regulated, and 246 circular transcripts (69.7%) were significantly down-regulated in tumor samples (Figure 3F). We also conducted a hierarchical cluster analysis to reveal the differential expression of full-length circRNA transcripts across tumor and normal samples. The tumor and the adjacent normal samples could be accurately classified into two different branches (Figure 4).

**FIGURE 5:**
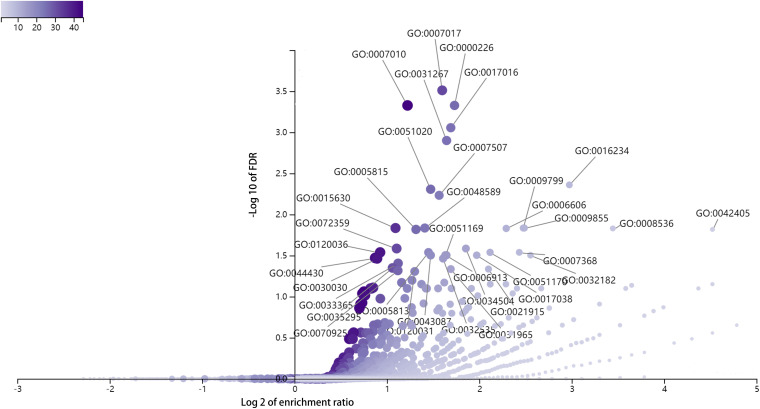
GO enrichment analysis of parental genes corresponding to differentially expressed circRNA full-length transcripts in cervical cancer.

To explore the biological functions of DE full-length circRNA transcripts in cervical cancer, we performed GO enrichment and KEGG pathway analyses of the parental genes corresponding to differential circRNA transcripts (Figure 5). The GO Biological Process enrichment analysis revealed that the parental genes were primarily associated with microtubule-based processes (GO:000701, *p* = 2.47E-08), microtubule cytoskeleton organization (GO:0000226, *p* = 9.30E-08), and cytoskeleton organization (GO:0007010, *p* = 1.13E-07). GO Cellular Components enrichment analysis revealed that parental genes were primarily associated with inclusion body (GO:0016234, *p* = 2.10E-06), microtubule cytoskeleton (GO:0015630, *p* = 1.40E-06), and nuclear inclusion body (GO:0042405, *p* = 1.95E-05). The GO Molecular Function enrichment analysis revealed that the parental genes were primarily associated with Ras GTPase binding (GO:0017016, *p* = 2.82E-07), small GTPase binding (GO:0031267, *p* = 5.05E-07), and ubiquitin-like protein binding (GO:0032182, *p* = 6.67E-05) functions. In addition, there was no significant enrichment in the KEGG signaling pathway (FDR > 0.05).

### Construction of the circRNA-miRNA Regulatory Network in Cervical Cancer

Previous studies have shown that circRNA can compete with miRNA and regulate miRNA-mediated downstream targeted gene expression in a variety of diseases. However, previous studies have failed to reveal the internal composition of circRNA; consequently, our understanding of the interaction between miRNA and circRNA containing multiple exons remains incomplete.

**FIGURE 6:**
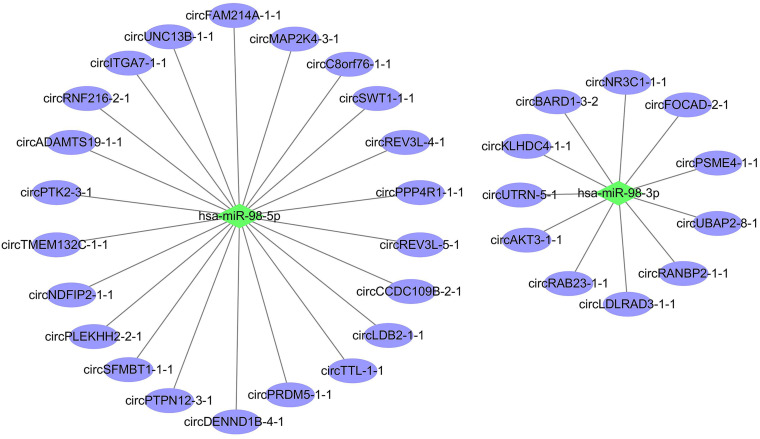
CircRNA-miRNA co-expression regulatory network in cervical cancer. The green and purple nodes represent miRNAs and circRNA full-length transcripts, respectively. Edge denotes their relationship.

Regulatory networks are an important tool for studying cancer, including cervical cancer (Song et al., 2018; Yi et al., 2019). We constructed a circRNA-miRNA co-expression regulatory network based on the DE full-length circRNA transcripts to further explore the potential biological functions of circRNA in cervical cancer. First, we employed a dataset from the HMDD (Human microRNA Disease Database) (Huang et al., 2019), which contained the experimentally DE miRNAs in cervical cancer. A total of 145 miRNAs were collected. Next, miRanda and TargetScan pipelines were used to predict the interaction between DE full-length circRNA transcripts and DE miRNAs in cervical cancer (see details in Materials and Methods). Finally, a total of 33 interaction pairs were obtained, and the circRNA-miRNA regulatory network was constructed (Figure 6). Our analysis demonstrated that miR-98-5p can interact with 22 circRNA transcripts, and miR-98-3p can interact with 11 circRNA transcripts. This finding suggested that the biological function of has-miR-98 during the development of cervical cancer is to act as a regulatory mediator of DE circRNA transcripts.

### Construction of the circRNA-miRNA-mRNA Regulatory Network in Cervical Cancer

To further explore the downstream target genes regulated by miR-98-5p and miR-98-3p in cervical cancer, we collected 4 cervical cancer patient tumors and adjacent normal tissue samples for total RNA sequencing. HISAT2 and StringTie pipelines were used to determine mRNA transcript expression in cervical cancer (see details in “Materials and Methods”). A total of 169,739 linear mRNA transcripts were identified in all samples, of which 149,838 transcripts were annotated in gene transfer format file (GTF, GRCh37/hg19, downloaded from Ensembl Genome Browser) and 19,901 transcripts were newly discovered. Based on read counts, we used DESeq2 to identify the DE mRNA transcripts. We made volcano plots for mRNA transcripts in cervical cancer-paired samples (| log2 (fold change) | > 1, adjusted *p*-value < 0.05) (Supplementary Figure S1A). In total, 881 mRNA transcript isoforms were found to be DE in cervical cancer, of which 421 (47.8%) transcript isoforms were up-regulated and 460 (52.2%) transcript isoforms were down-regulated in tumor samples. Cluster analysis based on the differential expression of mRNA transcripts across tumor and normal samples showed that the tumor and the adjacent normal samples could be accurately classified into two different branches (Supplementary Figure S1B).

**FIGURE 7:**
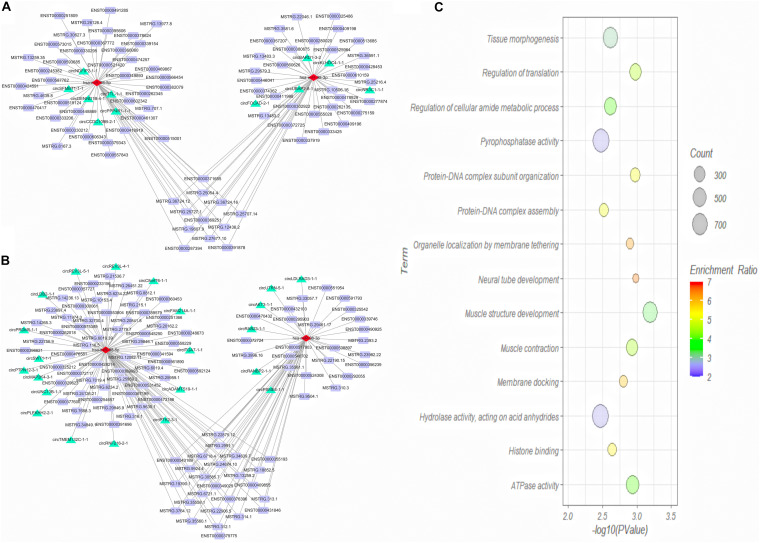
Competing endogenous RNA (ceRNA) regulatory network **(A,B)** and GO analysis of target genes **(C)** in cervical cancer. **(A)** Up-regulated ceRNA network; **(B)** Down-regulated ceRNA network. Red, green and purple nodes represent differentially expressed miRNAs, circRNA full-length transcripts and mRNA transcripts, respectively. Edge denotes their relationship. **(C)** GO enrichment analysis of genes corresponding to differentially expressed mRNA transcripts in network.

We then predicted the interaction between DE miRNAs and DE mRNA transcript isoforms in cervical cancer, and a total of 226 miRNA-mRNA interaction pairs were obtained. By combining the interaction pairs in the circRNA-miRNA regulatory network and positive relationship with circRNA and mRNA, two circRNA-miRNA-mRNA competitive regulatory networks with different regulated trends were then constructed (Figures 7A,B). One network included 11 up-regulated full-length circRNA transcripts, 2 miRNAs, and 82 up-regulated mRNA transcripts (Figure 7A). Another network included 22 down-regulated full-length circRNA transcripts, 2 miRNAs, and 107 down-regulated mRNA transcripts (Figure 7B).

To explore the function of aberrantly expressed mRNA transcripts in the ceRNA network, the annotated genes corresponding to the mRNA transcripts were analyzed by WebGestalt. We analyzed the functions of a total of 105 DE genes (Figure 7C). GO Biological Process term enrichment analysis showed that the target genes in the ceRNA network were enriched in muscle structure development, neural tube development, regulation of translation, protein-DNA complex subunit organization, and muscle contraction (*P* < 0.05). The GO Molecular Function term analysis revealed that the DE genes were enriched in ATPase activity, histone binding, pyrophosphatase activity, and hydrolase activity (*P* < 0.05).

### Overall Survival of Target Genes in the Network

**FIGURE 8:**
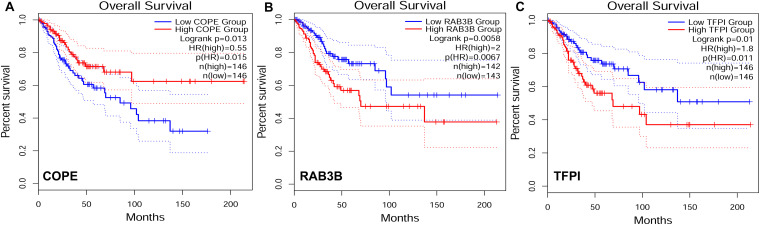
Target genes correlated to survival curves with significance in competing endogenous RNA (ceRNA) network. HR, hazard ratio. **(A)** The gene expression was negatively correlated to OS. **(B–C)** The gene expression was positively correlated to OS.

To explore the relationship between our identified target genes and clinical features, we utilized the Gene Expression Profiling Interactive Analysis (GEPIA) database. A total of 3 out of 105 DE genes from the ceRNA network were significantly correlated with CESC OS (*P* < 0.05) (Figure 8). The expression level of *COPE* was negatively correlated to OS (i.e., survival was longer for patients with higher expression levels in CESC). The expression level of *RAB3B* and *TFPI* were positively correlated to OS (i.e., survival was shorter for patients with higher expression levels in CESC).

## Discussion

Cervical cancer is the fourth most common cancer and the fourth main cause of cancer mortality in women worldwide (Bray et al., 2018). Although previous studies have reported that HPV plays a critical role in the development of cervical cancer (Walboomers et al., 1999), the specific molecular mechanism following viral infection remains unclear. Previous studies have shown that non-coding RNA, such as miRNAs (Liu et al., 2018; Pardini et al., 2018) and lncRNAs (Bhan et al., 2017; Peng et al., 2017; Luan and Wang, 2018), participate in various biological processes of cervical cancer. Recent work shows that circular RNA (circRNA), a new type of non-coding RNA with a special structure, also plays an important role in cancer. The functions of most circRNAs are thought to include acting as miRNA sponges (Hansen et al., 2013a, b; Memczak et al., 2013), interacting with RNA-binding proteins (Ashwal-Fluss et al., 2014; Dudekula et al., 2016), and translating peptides (Legnini et al., 2017; Pamudurti et al., 2017; Zhang et al., 2018). Previous studies have shown that circRNA has the potential to compete with endogenous RNA in cervical cancer. However, as in RNAs formed by linear splicing, alternative splicing events also occur in circRNAs and can lead to the production of multiple transcripts (Gao et al., 2016; Zhang X.-O. et al., 2016; Wu et al., 2019). Few studies have examined the expression of full-length circRNA transcripts in cervical cancer. To address this knowledge gap, we systematically explored the DE full-length circRNA transcripts and more precisely predicted the regulation target sites of circRNA in cervical cancer.

We characterized a total of 14,125 circRNAs in cervical cancer and successfully assembled 9,359 full-length transcripts of circRNA derived from exons. Furthermore, we found that 81% of circRNAs had only one circular transcript isoform, indicating that most exon-derived circRNAs were not derived from alternative splicing events in cervical cancer. Nevertheless, 2% of circRNAs contained more than 3 circular transcript isoforms. The length of full-length circRNA transcripts formed by one exon (median value was 404.5 bp) was significantly higher than that of circular transcripts composed of other numbers of exons (median value was 248.1 bp, *p* < 0.05). Furthermore, 353 full-length circRNA transcripts and 881 lineal mRNA transcripts were identified to be DE in tumors and adjacent normal tissues. The circRNA-miRNA-mRNA regulatory network was also established in our study. The network included 33 DE circRNA transcripts, 2 DE miRNAs, and 189 DE mRNA transcripts in cervical cancer. Analysis of a TCGA dataset revealed that a total of 3 target genes (*COPE*, *RAB3B*, and *TFPI*) in the regulatory network were correlated to survival curves with clinically significant outcomes in cervical cancer.

Previous studies have reported the overexpression of *RAB3B* in various types of cancer, such as laryngeal squamous cell carcinoma (Zhao L. et al., 2019) and lung squamous cell carcinoma (Zhang C. et al., 2016). In addition, overexpression of *RAB3B* has been associated with the prognosis, clinicopathological features, and the proliferation ability of tumor cells (Liu et al., 2014; Ye et al., 2014). All of these results thus indicate that *RAB3B* is an oncogene. By further analysis, there are 33 full-length circRNA transcripts associated with *RAB3B*. We suspect that circRNA may be involved in the development of cervical cancer by regulating the expression of *RAB3B*; however, additional experiments of regulation pairs are needed to confirm this speculation.

In sum, our work provided a detailed list of changes in the expression of full-length circRNA transcripts in cervical cancer. This list of circRNAs may potentially serve as a novel biomarker or therapeutic target. The ceRNA network also provides potential targets for future functional studies of circRNAs in cervical cancer.

## Data Availability Statement

The datasets presented in this study can be found in online repositories. The names of the repository/repositories and accession number(s) can be found in the article/Supplementary Material.

## Ethics Statement

The studies involving human participants were reviewed and approved by the Ethics Committee of The First Affiliated Hospital with Nanjing Medical University (No. 2020-SR-387). The patients/participants provided their written informed consent to participate in this study.

## Author Contributions

TX conceived and designed the study, collected the public data, performed the analysis, and wrote the manuscript. XS assisted with manuscript review and revision. YW provided the technical support and performed the analysis. SF and PH collected the clinical samples and assisted with manuscript review. All authors read and approved the final manuscript.

## Conflict of Interest

The authors declare that the research was conducted in the absence of any commercial or financial relationships that could be construed as a potential conflict of interest.

## References

[B1] Agarwal V. Bell G. W. Nam J. W. Bartel D. P. (2015). Predicting effective microRna target sites in mammalian mRnas. *eLife* 4:e05005. 10.7554/eLife.05005 26267216 PMC4532895

[B2] Ashwal-Fluss R. Meyer M. Pamudurti N. R. Ivanov A. Bartok O. Hanan M. (2014). circRNA biogenesis competes with pre-mRNA splicing. *Mol. Cell* 56 55–66. 10.1016/j.molcel.2014.08.019 25242144

[B3] Betel D. Koppal A. Agius P. Sander C. Leslie C. (2010). Comprehensive modeling of microRNA targets predicts functional non-conserved and non-canonical sites. *Genome Biol.* 11:R90. 10.1186/gb-2010-11-8-r90 20799968 PMC2945792

[B4] Bhan A. Soleimani M. Mandal S. S. (2017). Long noncoding RNA and cancer: a new paradigm. *Cancer Res.* 77 3965–3981. 10.1158/0008-5472.CAN-16-2634 28701486 PMC8330958

[B5] Bray F. Ferlay J. Soerjomataram I. Siegel R. L. Torre L. A. Jemal A. (2018). Global cancer statistics 2018: GLOBOCAN estimates of incidence and mortality worldwide for 36 cancers in 185 countries. *CA Cancer J. Clin.* 68 394–424. 10.3322/caac.21492 30207593

[B6] Chen S. Huang V. Xu X. Livingstone J. Soares F. Jeon J. (2019). Widespread and Functional RNA circularization in localized prostate cancer. *Cell* 176 831–843.e822. 10.1016/j.cell.2019.01.025 30735634

[B7] Chen W. Zheng R. Baade P. D. Zhang S. Zeng H. Bray F. (2016). Cancer statistics in China, 2015. *CA Cancer J. Clin.* 66 115–132. 10.3322/caac.21338 26808342

[B8] Cohen P. A. Jhingran A. Oaknin A. Denny L. (2019). Cervical cancer. *Lancet* 393 169–182. 10.1016/S0140-6736(18)32470-X 30638582

[B9] Dudekula D. B. Panda A. C. Grammatikakis I. De S. Abdelmohsen K. Gorospe M. (2016). CircInteractome: a web tool for exploring circular RNAs and their interacting proteins and microRNAs. *RNA Biol.* 13 34–42. 10.1080/15476286.2015.1128065 26669964 PMC4829301

[B10] Gao Y. Wang J. Zheng Y. Zhang J. Chen S. Zhao F. (2016). Comprehensive identification of internal structure and alternative splicing events in circular RNAs. *Nat. Commun.* 7:12060. 10.1038/ncomms12060 27350239 PMC4931246

[B11] Gao Y. Zhang J. Zhao F. (2018). Circular RNA identification based on multiple seed matching. *Brief. Bioinform.* 19 803–810. 10.1093/bib/bbx014 28334140

[B12] Han D. Li J. Wang H. Su X. Hou J. Gu Y. (2017). Circular RNA circMTO1 acts as the sponge of microRNA-9 to suppress hepatocellular carcinoma progression. *Hepatology* 66 1151–1164. 10.1002/hep.29270 28520103

[B13] Hansen T. B. Jensen T. I. Clausen B. H. Bramsen J. B. Finsen B. Damgaard C. K. (2013a). Natural RNA circles function as efficient microRNA sponges. *Nature* 495 384–388. 10.1038/nature11993 23446346

[B14] Hansen T. B. Kjems J. Damgaard C. K. (2013b). Circular RNA and miR-7 in cancer. *Cancer Res.* 73 5609–5612. 10.1158/0008-5472.CAN-13-1568 24014594

[B15] Heng L. (2013). Aligning sequence reads, clone sequences and assembly contigs with BWA-MEM. *arXiv* [Preprint], Available online at: https://arxiv.org/abs/1303.3997

[B16] Hirsch S. Blätte T. J. Grasedieck S. Cocciardi S. Rouhi A. Jongen-Lavrencic M. (2017). Circular RNAs of the nucleophosmin (NPM1) gene in acute myeloid leukemia. *Haematologica* 102 2039–2047. 10.3324/haematol.2017.172866 28971903 PMC5709103

[B17] Huang Z. Shi J. Gao Y. Cui C. Zhang S. Li J. (2019). HMDD v3.0: a database for experimentally supported human microRNA-disease associations. *Nucleic Acids Res.* 47 D1013–D1017. 10.1093/nar/gky1010 30364956 PMC6323994

[B18] Jeck W. R. Sharpless N. E. (2014). Detecting and characterizing circular RNAs. *Nat. Biotechnol.* 32 453–461. 10.1038/nbt.2890 24811520 PMC4121655

[B19] Kim D. Langmead B. Salzberg S. L. (2015). HISAT: a fast spliced aligner with low memory requirements. *Nat. Methods* 12 357–360. 10.1038/nmeth.3317 25751142 PMC4655817

[B20] Kozomara A. Birgaoanu M. Griffiths-Jones S. (2019). miRBase: from microRNA sequences to function. *Nucleic Acids Res.* 47 D155–D162. 10.1093/nar/gky1141 30423142 PMC6323917

[B21] Kristensen L. S. Hansen T. B. Venø M. T. Kjems J. (2018). Circular RNAs in cancer: opportunities and challenges in the field. *Oncogene* 37 555–565. 10.1038/onc.2017.361 28991235 PMC5799710

[B22] Legnini I. Di Timoteo G. Rossi F. Morlando M. Briganti F. Sthandier O. (2017). Circ-ZNF609 is a circular RNA that can be translated and functions in myogenesis. *Mol. Cell* 66 22–37.e9. 10.1016/j.molcel.2017.02.017 28344082 PMC5387670

[B23] Li P. Chen H. Chen S. Mo X. Li T. Xiao B. (2017). Circular RNA 0000096 affects cell growth and migration in gastric cancer. *Br. J. Cancer* 116 626–633. 10.1038/bjc.2016.451 28081541 PMC5344286

[B24] Li X. Liu C. Xue W. Zhang Y. Jiang S. Yin Q. (2017). Coordinated circRNA biogenesis and function with NF90/NF110 in viral infection. *Mol. Cell* 67 214–227.e7. 10.1016/j.molcel.2017.05.023 28625552

[B25] Li S. Teng S. Xu J. Su G. Zhang Y. Zhao J. (2019). Microarray is an efficient tool for circRNA profiling. *Brief. Bioinform.* 20 1420–1433. 10.1093/bib/bby006 29415187

[B26] Li Z. Huang C. Bao C. Chen L. Lin M. Wang X. (2015). Exon-intron circular RNAs regulate transcription in the nucleus. *Nat. Struct. Mol. Biol.* 22 256–264. 10.1038/nsmb.2959 25664725

[B27] Liao Y. Wang J. Jaehnig E. J. Shi Z. Zhang B. (2019). WebGestalt 2019: gene set analysis toolkit with revamped UIs and APIs. *Nucleic Acids Res.* 47 W199–W205. 10.1093/nar/gkz401 31114916 PMC6602449

[B28] Liu Q. Tang H. Liu X. Liao Y. Li H. Zhao Z. (2014). miR-200b as a prognostic factor targets multiple members of RAB family in glioma. *Med. Oncol.* 31:859. 10.1007/s12032-014-0859-x 24477653

[B29] Liu Q. Zhang X. Hu X. Dai L. Fu X. Zhang J. (2016). Circular RNA related to the chondrocyte ECM regulates MMP13 expression by functioning as a MiR-136 ‘Sponge’ in human cartilage degradation. *Sci. Rep.* 6:22572. 10.1038/srep22572 26931159 PMC4773870

[B30] Liu S. S. Chan K. K. L. Chu D. K. H. Wei T. N. Lau L. S. K. Ngu S. F. (2018). Oncogenic microRNA signature for early diagnosis of cervical intraepithelial neoplasia and cancer. *Mol. Oncol.* 12 2009–2022. 10.1002/1878-0261.12383 30221475 PMC6275249

[B31] Love M. I. Huber W. Anders S. (2014). Moderated estimation of fold change and dispersion for RNA-seq data with DESeq2. *Genome Biol.* 15:550. 10.1186/s13059-014-0550-8 25516281 PMC4302049

[B32] Luan X. Wang Y. (2018). LncRNA XLOC_006390 facilitates cervical cancer tumorigenesis and metastasis as a ceRNA against miR-331-3p and miR-338-3p. *J. Gynecol. Oncol.* 29:e95. 10.3802/jgo.2018.29.e95 30207103 PMC6189437

[B33] Martin M. (2011). Cutadapt removes adapter sequences from high-throughput sequencing reads. *EMBnet J.* 17 10–12. 10.14806/ej.17.1.200

[B34] Memczak S. Jens M. Elefsinioti A. Torti F. Krueger J. Rybak A. (2013). Circular RNAs are a large class of animal RNAs with regulatory potency. *Nature* 495 333–338. 10.1038/nature11928 23446348

[B35] Pamudurti N. R. Bartok O. Jens M. Ashwal-Fluss R. Stottmeister C. Ruhe L. (2017). Translation of CircRNAs. *Mol. Cell* 66 9–21.e7. 10.1016/j.molcel.2017.02.021 28344080 PMC5387669

[B36] Pardini B. De Maria D. Francavilla A. Di Gaetano C. Ronco G. Naccarati A. (2018). MicroRNAs as markers of progression in cervical cancer: a systematic review. *BMC Cancer* 18:696. 10.1186/s12885-018-4590-4 29945565 PMC6020348

[B37] Peng W. X. Koirala P. Mo Y. Y. (2017). LncRNA-mediated regulation of cell signaling in cancer. *Oncogene* 36 5661–5667. 10.1038/onc.2017.184 28604750 PMC6450570

[B38] Pertea M. Pertea G. M. Antonescu C. M. Chang T.-C. Mendell J. T. Salzberg S. L. (2015). StringTie enables improved reconstruction of a transcriptome from RNA-seq reads. *Nat. Biotechnol.* 33 290–295. 10.1038/nbt.3122 25690850 PMC4643835

[B39] Piwecka M. Glažar P. Hernandez-Miranda L. R. Memczak S. Wolf S. A. Rybak-Wolf A. (2017). Loss of a mammalian circular RNA locus causes miRNA deregulation and affects brain function. *Science* 357:eaam8526. 10.1126/science.aam8526 28798046

[B40] Salzman J. Chen R. E. Olsen M. N. Wang P. L. Brown P. O. (2013). Cell-type specific features of circular RNA expression. *PLoS Genet.* 9:e1003777. 10.1371/journal.pgen.1003777 24039610 PMC3764148

[B41] Shannon P. Markiel A. Ozier O. Baliga N. S. Wang J. T. Ramage D. (2003). Cytoscape: a software environment for integrated models of biomolecular interaction networks. *Genome Res.* 13 2498–2504. 14597658 10.1101/gr.1239303 PMC403769

[B42] Song J. Ye A. Jiang E. Yin X. Chen Z. Bai G. (2018). Reconstruction and analysis of the aberrant lncRNA-miRNA-mRNA network based on competitive endogenous RNA in CESC. *J. Cell. Biochem.* 119 6665–6673. 10.1002/jcb.26850 29741786 PMC6055788

[B43] Song T. Xu A. Zhang Z. Gao F. Zhao L. Chen X. (2019). CircRNA hsa_circRNA_101996 increases cervical cancer proliferation and invasion through activating TPX2 expression by restraining miR-8075. *J. Cell. Physiol.* 234 14296–14305. 10.1002/jcp.28128 30633364 PMC13483104

[B44] Tang Q. Chen Z. Zhao L. Xu H. (2019). Circular RNA hsa_circ_0000515 acts as a miR-326 sponge to promote cervical cancer progression through up-regulation of ELK1. *Aging* 11 9982–9999. 10.18632/aging.102356 31772143 PMC6914414

[B45] Tang Z. Li C. Kang B. Gao G. Li C. Zhang Z. (2017). GEPIA: a web server for cancer and normal gene expression profiling and interactive analyses. *Nucleic Acids Res.* 45 W98–W102. 10.1093/nar/gkx247 28407145 PMC5570223

[B46] Tornesello M. L. Faraonio R. Buonaguro L. Annunziata C. Starita N. Cerasuolo A. (2020). The role of microRNAs, long non-coding RNAs, and circular RNAs in cervical cancer. *Front. Oncol.* 10:150. 10.3389/fonc.2020.00150 32154165 PMC7044410

[B47] Vo J. N. Cieslik M. Zhang Y. Shukla S. Xiao L. Zhang Y. (2019). The landscape of circular RNA in cancer. *Cell* 176 869–881.e813. 10.1016/j.cell.2018.12.021 30735636 PMC6601354

[B48] Walboomers J. M. Jacobs M. V. Manos M. M. Bosch F. X. Kummer J. A. Shah K. V. (1999). Human papillomavirus is a necessary cause of invasive cervical cancer worldwide. *J. Pathol.* 189 12–19. 10451482 10.1002/(SICI)1096-9896(199909)189:1<12::AID-PATH431>3.0.CO;2-F

[B49] Wang Y. Song F. Zhu J. Zhang S. Yang Y. Chen T. (2017). GSA: genome sequence archive. *Genomics Proteom. Bioinform.* 15 14–18. 10.1016/j.gpb.2017.01.001 28387199 PMC5339404

[B50] Wu J. Li Y. Wang C. Cui Y. Xu T. Wang C. (2019). CircAST: full-length assembly and quantification of alternatively spliced isoforms in circular RNAs. *Genomics Proteom. Bioinform.* 17 522–534. 10.1016/j.gpb.2019.03.004 32007626 PMC7056934

[B51] Xie H. Ren X. Xin S. Lan X. Lu G. Lin Y. (2016). Emerging roles of circRNA_001569 targeting miR-145 in the proliferation and invasion of colorectal cancer. *Oncotarget* 7 26680–26691. 10.18632/oncotarget.8589 27058418 PMC5042007

[B52] Xu T. Wu J. Han P. Zhao Z. Song X. (2017). Circular RNA expression profiles and features in human tissues: a study using RNA-seq data. *BMC Genomics* 18 (Suppl. 6):680. 10.1186/s12864-017-4029-3 28984197 PMC5629547

[B53] Ye F. Tang H. Liu Q. Xie X. Wu M. Liu X. (2014). miR-200b as a prognostic factor in breast cancer targets multiple members of RAB family. *J. Transl. Med.* 12:17. 10.1186/1479-5876-12-17 24447584 PMC3898994

[B54] Yi Y. Liu Y. Wu W. Wu K. Zhang W. (2019). Reconstruction and analysis of circRNA-miRNA-mRNA network in the pathology of cervical cancer. *Oncol. Rep.* 41 2209–2225. 10.3892/or.2019.7028 30816541 PMC6412533

[B55] Yu C.-Y. Li T.-C. Wu Y.-Y. Yeh C.-H. Chiang W. Chuang C.-Y. (2017). The circular RNA circBIRC6 participates in the molecular circuitry controlling human pluripotency. *Nat. Commun.* 8:1149. 10.1038/s41467-017-01216-w 29074849 PMC5658440

[B56] Yu L. Gong X. Sun L. Zhou Q. Lu B. Zhu L. (2016). The circular RNA Cdr1as act as an oncogene in hepatocellular carcinoma through targeting miR-7 expression. *PLoS One* 11:e0158347. 10.1371/journal.pone.0158347 27391479 PMC4938625

[B57] Zhang C. Min L. Zhang L. Ma Y. Yang Y. Shou C. (2016). Combined analysis identifies six genes correlated with augmented malignancy from non-small cell to small cell lung cancer. *Tumour Biol.* 37 2193–2207. 10.1007/s13277-015-3938-5 26349752

[B58] Zhang X.-O. Dong R. Zhang Y. Zhang J.-L. Luo Z. Zhang J. (2016). Diverse alternative back-splicing and alternative splicing landscape of circular RNAs. *Genome Res.* 26 1277–1287. 10.1101/gr.202895.115 27365365 PMC5052039

[B59] Zhang J. Liu H. Hou L. Wang G. Zhang R. Huang Y. (2017). Circular RNA_LARP4 inhibits cell proliferation and invasion of gastric cancer by sponging miR-424-5p and regulating LATS1 expression. *Mol. Cancer* 16:151. 10.1186/s12943-017-0719-3 28893265 PMC5594516

[B60] Zhang M. Zhao K. Xu X. Yang Y. Yan S. Wei P. (2018). A peptide encoded by circular form of LINC-PINT suppresses oncogenic transcriptional elongation in glioblastoma. *Nat. Commun.* 9:4475. 10.1038/s41467-018-06862-2 30367041 PMC6203777

[B61] Zhao J. Lee E. E. Kim J. Yang R. Chamseddin B. Ni C. (2019). Transforming activity of an oncoprotein-encoding circular RNA from human papillomavirus. *Nat. Commun.* 10:2300. 10.1038/s41467-019-10246-5 31127091 PMC6534539

[B62] Zhao L. Cao H. Chi W. Meng W. Cui W. Guo W. (2019). Expression profile analysis identifies the long non-coding RNA landscape and the potential carcinogenic functions of LINC00668 in laryngeal squamous cell carcinoma. *Gene* 687 47–55. 10.1016/j.gene.2018.11.020 30415008

