# Supplementary material for: Genome-Wide Analysis of the Expression of Circular RNA Full-Length Transcripts and Construction of the circRNA-miRNA-mRNA Network in Cervical Cancer
Source: Front Cell Dev Biol. 2020 Nov 24;8:603516. doi: 10.3389/fcell.2020.603516 (PMC7732672; doi:10.3389/fcell.2020.603516)
Supplement: Supplementary file 1 [file Table_1.DOCX]

**Supplementary Information**

**Supplemental Figure S1.** Analysis of mRNA transcripts expression in cervical cancer. **A.** Volcano plot showing differentially expressed mRNA transcripts in cervical cancer. Red points and green points represent significantly up-regulated and down-regulated mRNA transcripts, respectively; **B.** Cluster analysis of differentially expressed mRNA transcripts in tumors and adjacent normal tissues in cervical cancer.

**Supplemental Table S1.** The number of circRNA full-length transcripts and linear mRNA transcripts distribution in different chromosomes in cervical cancer

| Chromosome | Circular transcript | | Linear transcript | | Proportion |
| --- | --- | --- | --- | --- | --- |
| chr1 | 957 | 17531 | | 5.46% | |
| chr2 | 842 | 13979 | | 6.02% | |
| chr3 | 687 | 11759 | | 5.84% | |
| chr4 | 443 | 7803 | | 5.68% | |
| chr5 | 480 | 9107 | | 5.27% | |
| chr6 | 458 | 8443 | | 5.42% | |
| chr7 | 479 | 9457 | | 5.07% | |
| chr8 | 399 | 7751 | | 5.15% | |
| chr9 | 368 | 6513 | | 5.65% | |
| chr10 | 448 | 6503 | | 6.89% | |
| chr11 | 452 | 12204 | | 3.70% | |
| chr12 | 493 | 11387 | | 4.33% | |
| chr13 | 255 | 3022 | | 8.44% | |
| chr14 | 331 | 7522 | | 4.40% | |
| chr15 | 447 | 7314 | | 6.11% | |
| chr16 | 314 | 9697 | | 3.24% | |
| chr17 | 440 | 12580 | | 3.50% | |
| chr18 | 252 | 3578 | | 7.04% | |
| chr19 | 205 | 12758 | | 1.61% | |
| chr20 | 210 | 4040 | | 5.20% | |
| chr21 | 104 | 2178 | | 4.78% | |
| chr22 | 151 | 4459 | | 3.39% | |
| chrX | 143 | 6001 | | 2.38% | |
