# Supplementary figures and images for: Genome-Wide Analysis of the Expression of Circular RNA Full-Length Transcripts and Construction of the circRNA-miRNA-mRNA Network in Cervical Cancer
Source: Front Cell Dev Biol. 2020 Nov 24;8:603516. doi: 10.3389/fcell.2020.603516 (PMC7732672; doi:10.3389/fcell.2020.603516)

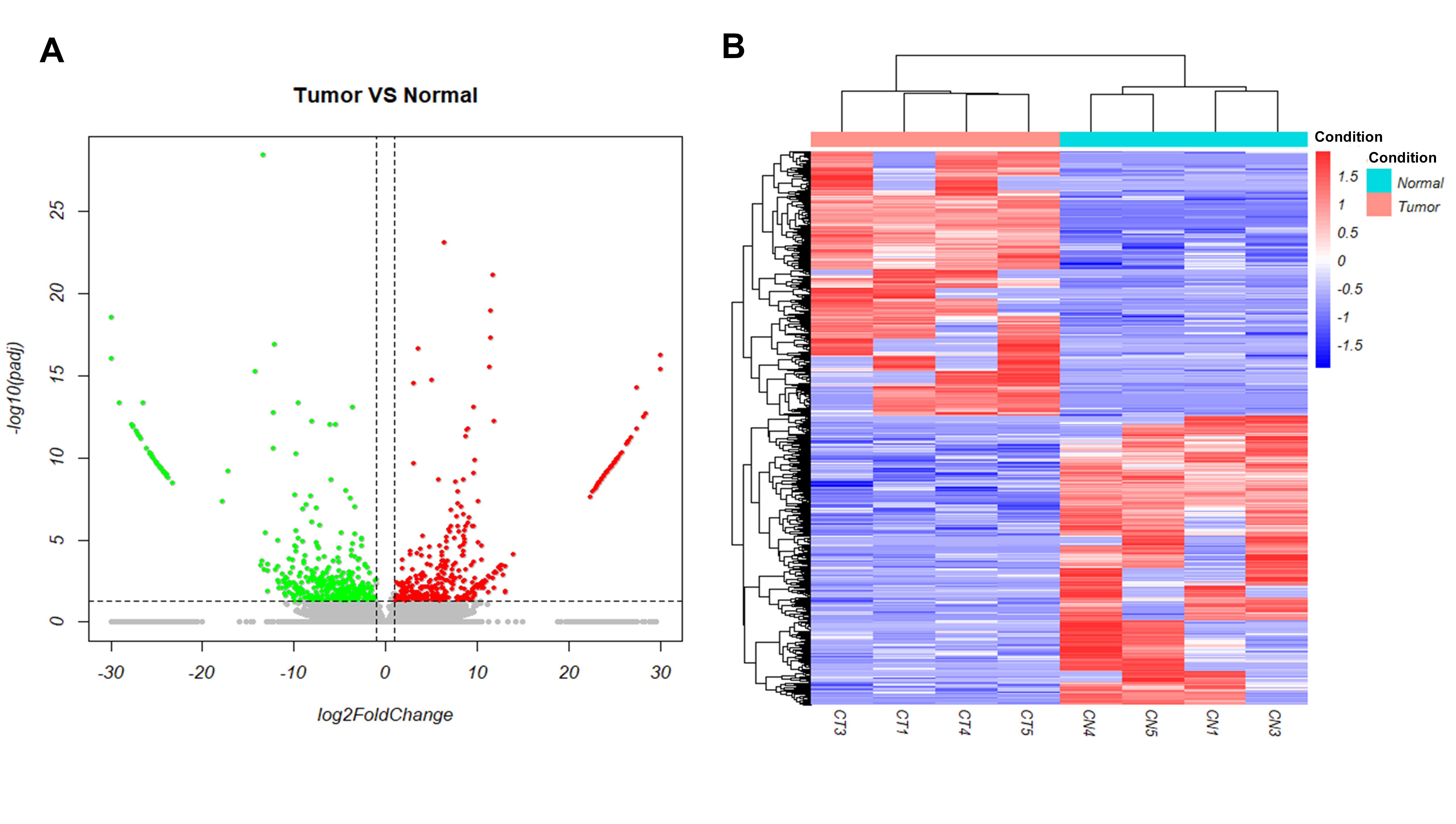

Supplement: Supplementary file 2 [file Image_1.TIF]
